# Human lactoferrin but not lysozyme neutralizes HSV-1 and inhibits HSV-1 replication and cell-to-cell spread

**DOI:** 10.1186/1743-422X-6-53

**Published:** 2009-05-12

**Authors:** Hannamari Välimaa, Jorma Tenovuo, Matti Waris, Veijo Hukkanen

**Affiliations:** 1 Institute of Dentistry, University of Turku, Turku, Finland; 2 Department of Virology, University of Turku, Turku, Finland; 3 Haartman Institute, Department of Virology, University of Helsinki, Helsinki, Finland; 4 Helsinki University Central Hospital Laboratory, Helsinki, Finland; 5 Department of Oral Diseases, Turku University Central Hospital, Turku, Finland; 6 Department of Microbiology, University of Oulu, Oulu, Finland

## Abstract

The frequent oral shedding of herpes simplex virus type 1 (HSV-1) in the absence of clinical disease suggests that symptomatic HSV-1 recurrences may be inhibited by the mucosal environment. Indeed, saliva has been shown to contain substances with anti-HSV activity. In the current study, we investigated the anti-HSV-1 activity of human lactoferrin (hLf) and lysozyme (hLz), two highly cationic polypeptides of the mucosal innate defence system.

HLf blocked HSV-1 infection at multiple steps of the viral replication cycle, whereas lysozyme displayed no anti-HSV-1 activity. Preincubation of HSV-1 virions and presence of hLf during or after viral absorption period or for the entire HSV-1 infection cycle inhibited HSV-1 infection by reducing both the plaque count and plaque size in a dose- and virus strain-dependent manner. Cell-to-cell spread of wild-type HSV-1 and the strain gC-39, deleted of glycoprotein C, was dramatically reduced, but the cell-to-cell spread of HSV-1 Rid1, harboring a mutated gD and thus unable to react with the cellular HVEM receptor, remained unchanged. This suggests that the inhibition of cell-to-cell spread is mediated by effects on gD or its cellular counterparts.

Our results show that the cationic nature is not a major determinant in the anti-HSV action of mucosal innate cationic polypeptides, since whereas hLf inhibited HSV-1 infection efficiently, hLz had no HSV-1 inhibiting activity. Our results show that in addition to inhibiting the adsorption and post-attachment events of HSV-1 infection, hLf is also able to neutralize HSV-1 and that the inhibition of cell-to-cell spread involves viral gD. These results suggest that Lf may have a significant role in the modulation of HSV-1 infection in the oral cavity as well as in the genital mucosa, the major sites of HSV-1 infection.

## Findings

Oral mucosa is a common site of primary herpes simplex virus type 1 (HSV-1) infection. After a primary infection in the oral region, HSV-1 establishes latency in the trigeminal ganglion. Subsequent reactivation of HSV-1 typically results in asymptomatic shedding of virus into saliva, but only rarely in detectable intraoral lesions. Instead, extraoral manifestations are frequent and affect 20–40% of the HSV-seropositive population [1]. Thus the intraoral environment may inhibit replication of HSV-1.

Lactoferrin (Lf) and lysozyme (Lz) are highly cationic polypeptides with broad antimicrobial properties, abundant in saliva, tears and cervicovaginal mucosal fluid. The major inhibitory mechanism of Lf on HSV infection has been suggested to occur by competitive binding of Lf to negatively charged cell surface glycosaminoglycans (GAGs) [2-4]. Furthermore, we have observed human Lf (hLf) to inhibit post-attachment events in the HSV-1 replication including entry and cell-to-cell spread [5]. This finding has been confirmed with both bovine and human Lf [6,7]. Lactoferrin has also been suggested to affect the HSV reactivation phenotype in the oral region [8].

The most characterized antimicrobial property of Lz is its antibacterial muramidase activity, although some enzyme-independent functions have been reported [9]. Observations on anti-HSV activity of Lz are contradictory [10,11], but likely involve both enzymatic and cationic properties of this enzyme [10].

In this study, we compared the anti-HSV activity of human lactoferrin and lysozyme, and the roles of viral glycoproteins gC and gD therein. We infected Vero cell monolayers with HSV-1 in the presence or absence of human milk-derived non-iron-saturated lactoferrin (apoLf; L-0520, Sigma), iron-saturated lactoferrin (satLf; L-3770, Sigma) or lysozyme (hLz; L-6394, Sigma). Virus strains used were the wild-type (wt) HSV-1 strain KOS, and the KOS-derived mutants Rid1 and gC^-^39. Rid1 has a single amino acid substitution at position 27 of gD, a viral glycoprotein essential for viral entry, that results in the inability of this virus to use HVEM as an entry mediator [12]. gC^-^39 lacks glycoprotein C, the major viral envelope glycoprotein mediating initial attachment of HSV-1 to cell surface GAGs [13].

**Figure 1:**
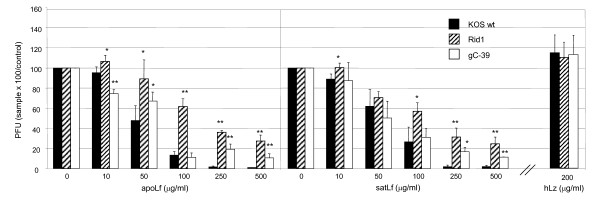
**Lactoferrin but not lysozyme inhibited efficiently HSV-1 infection of Vero cells**. Replication of wt HSV-1 and mutant viruses gC^-^39 and Rid1 in Vero cells with human non-iron-saturated Lf (apoLf), iron-saturated Lf (satLf) or lysozyme (hLz) present throughout the entire replication cycle. Lactoferrin and lysozyme were added to cell cultures 24 h prior to infection. The relative amount of viral plaques, in comparison to untreated cultures, is shown. Error bars represent standard deviations of three separate experiments. Statistical significance in the level of inhibition: * = p < 0.05, ** = p < 0.005, mutant viruses compared to wt KOS. Only results with the highest concentration of hLz used are shown.

**Figure 2:**
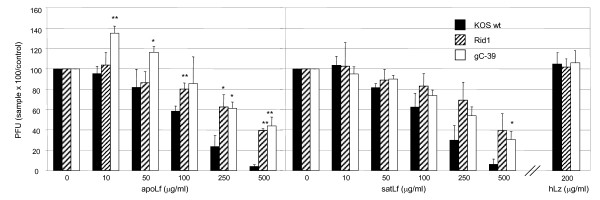
**Lactoferrin but not lysozyme neutralized HSV-1**. Wt HSV-1 and mutant viruses gC^-^39 and Rid1 were incubated for 30 min at 37°C with different concentrations of human non-iron-saturated Lf (apoLf), iron-saturated Lf (satLf) or lysozyme (hLz) prior to inoculation of Vero cell cultures. Preincubated virus/hLf mixtures were diluted 1:10 upon addition to the cell culture so that the inoculum contained 1–50 μg/ml of hLf. Therefore the observed inhibition could not have resulted from the hLf effect on the cells. The relative amount of viral plaques, in comparison to untreated cultures, is shown. Error bars represent standard deviations of three separate experiments. Statistical significance in the level of inhibition: * = p < 0.05, ** = p < 0.005, mutant viruses compared to wt KOS. Only results with the highest concentration of hLz used are shown.

**Figure 3:**
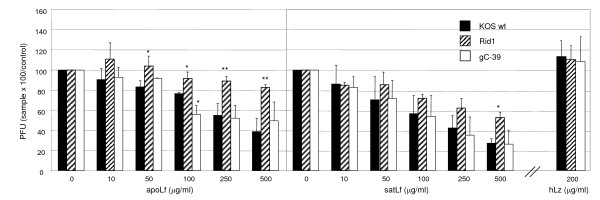
**Pretreatment of cell cultures with lactoferrin but not lysozyme inhibited HSV-1 infection**. Vero cell cultures were pretreated with human non-iron-saturated Lf (apoLf), iron-saturated Lf (satLf) or lysozyme (hLz) for 24 h prior to inoculation. To exclude lactoferrin and lysozyme effect on HSV-1 virion, these supplements were removed from the media by washing before inoculation of cultures with wt HSV-1 and mutant viruses gC^-^39 and Rid1. The relative amount of viral plaques, in comparison to untreated cultures, is shown. Error bars represent standard deviations of three separate experiments. Statistical significance in the level of inhibition: * = p < 0.05, ** = p < 0.005, mutant viruses compared to wt KOS. Only results with the highest concentration of hLz used are shown.

**Figure 4:**
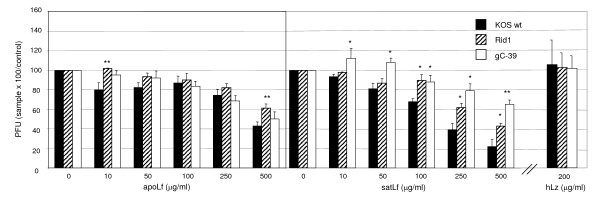
**Lactoferrin has inhibitory activity against HSV-1 infection also after viral adsorption**. To study the lactoferrin effect on post-attachment events, wt HSV-1 and mutant viruses gC^-^39 and Rid1 were adsorbed to cells at 4°C, cultures were washed and human non-iron-saturated Lf (apoLf), iron-saturated Lf (satLf) or lysozyme (hLz) was added. The relative amount of viral plaques, in comparison to untreated cultures, is shown. Error bars represent standard deviations of three separate experiments. Statistical significance in the level of inhibition: * = p < 0.05, ** = p < 0.005, mutant viruses compared to wt KOS. Only results with the highest concentration of hLz used are shown.

First, confluent Vero cell monolayers on 24-well plates were infected with 100 or 200 plaque-forming units (pfu) of HSV-1 KOS, Rid1 or gC^-^39. For the infection studies, fetal calf serum (FCS) concentration of MEM culture medium with antibiotics was reduced from 10% to 1% and medium was supplemented with 0–500 μg/ml of apoLf or satLf, or 0–200 μg/ml of hLz at various time points of infection (Figures 1, 2, 3, 4). Cultures with medium without hLf and hLz served as negative controls. Quadruplicate cultures were fixed with methanol 20 hours post-infection (h.p.i.), and infected cells were identified by immunoperoxidase staining (IPS) of viral proteins with HRP-labelled polyclonal antibodies against HSV-1 (P0175, DakoCytomation, Denmark, A/S) (modified from [14]). The number of plaques and plaque size was determined by light microscopy.

Second, 50 pfu of HSV-1 KOS, Rid1, or gC^-^39 was adsorbed to Vero cell monolayers in 12-well plates for 2 h at 37°C. The inoculum was removed and fresh MEM added, containing 1% FCS, antibiotics and 0.1% human gammaglobulin supplemented with 0–500 μg/ml of apoLf or satLf, or 0–200 μg/ml of hLz. After five days, plaques formed by viruses were visualized by crystal violet staining. Data were analysed by Student's two-tailed paired t-test, and p-values less than 0.05 were considered significant.

We found human lactoferrin (hLf) to interfere with the progression of HSV-1 infection at multiple steps of the viral replication cycle, whereas human lysozyme displayed no anti-HSV-1 activity. The degree of hLf iron-saturation played little role in HSV-1 inhibition.

In the series of inhibition assays, cells were first maintained in hLf containing medium for 24 h before viral inoculation and subsequently through the whole replication cycle (Figure 1). Already at a hLf concentration of 50–100 μg/ml, HSV-1 replication was dramatically inhibited independent of the degree of Lf iron-saturation. Both of the mutant viruses, Rid1 and gC^-^39, were significantly more resistant to hLf-mediated inhibition, indicating a role for both gC and gD in the inhibition of viral replication.

A series of further experiments was carried out investigating hLf effect on the virion, the absorption and post-absorption events in the replication cycle and cell-to-cell spread. We found hLf to neutralize unbound wt HSV-1 with an IC_50 _of 100–250 μg/ml of hLf (Figure 2). HSV-1 mutants, gC^-^39 and Rid1, were significantly more resistant to hLf-mediated neutralization, suggesting that neutralization is mediated by effects of hLf on gC and gD or their cellular counterparts. Moreover, preincubation of gC^-^39 with apoLf unexpectedly enhanced infectivity in low concentrations. This suggests that hLf binding to virion compensates for gC and facilitates formation of contacts between virion bound hLf and cell surface Lf receptors, as has been earlier also suggested to occur between bovine Lf and gC^-^39 [4]. Instead, bLf has been found unable to neutralize wt HSV-1 [3,6], although it has been shown to bind to wt HSV-1 [2].

Wt HSV-1 and gC^-^39 were equally sensitive (IC_50 _250 μg/ml) to hLf pretreatment of cells (Figure 3), suggesting that this inhibition involves interactions participated by both gC and gB, the other major glycoprotein mediating attachment [15]. Cell surface GAGs have been shown to bind Lf [4] and Lf anti-HSV-1 activity to decrease in the absence of heparan sulphate [3] or viral gC when Lf was added to cultures simultaneously with the virus [4]. These findings strongly suggest a major role for inhibition of initial attachment of both gB and gC to GAGs in the anti-HSV action of Lf. In our study, pretreatment of cells inhibited also Rid1 in higher hLf concentrations, suggesting that hLf inhibition is not restricted only to HSV attachment to the cell surface, but may also modulate gD-mediated viral entry.

The sensitivity of post-attachment steps to hLf-mediated inhibition was confirmed by the observed anti-HSV-1 activity of hLf added to cultures after viral adsorption (Figure 4). Of the studied viral strains, wt HSV-1 was the most sensitive with IC_50 _of 250–500 μg/ml of hLf. Iron-saturated form of hLf was slightly more effective. Rid1 infectivity decreased 40–60% at a hLf concentration of 500 μg/ml. Earlier, bLf has been shown not to affect differentially on the entry of wt HSV-1 and Rid1 strains in a β-galactosidase assay [3]. Therefore, the observed gD-associated post-attachment inhibition in our study may yield from Lf interference with intracellular events in the replication cycle or with the synthetized progeny viral components or virions. Previously, both hLf [5] and bLf [6] have been shown to reduce HSV-1 plaque number if added immediately after viral adsorption or 1 h after viral entry. This inhibition has been suggested to result from Lf binding to viral components intracellularly [6]. The N-terminal region of Lf has been shown to carry a nuclear localization signal [16] and to be able to translocate to the nucleus of the cell [17]. The efficiency and mechanisms of Lf binding and intake seem to be cell type-specific and to involve various receptors [3,7,17-19]. Therefore certain intracellular antiviral effects of Lf may differ between cell types.

**Figure 5:**
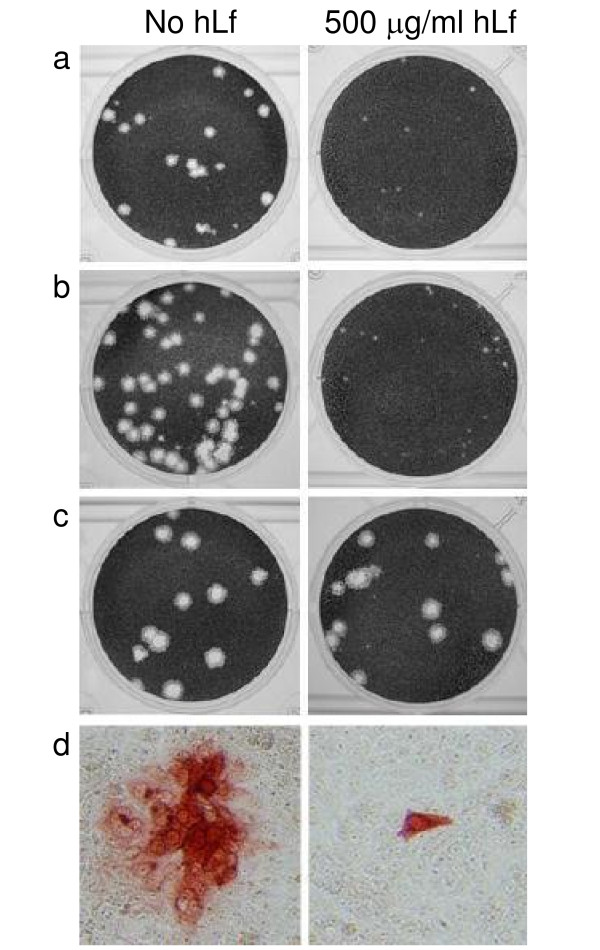
**Lactoferrin inhibition of HSV-1 cell-to-cell spread is mediated by gD**. HSV-1 KOS (a, d), gC^-^39 (b) and Rid1 (c) infection of Vero cells in the absence or presence of 500 μg/ml of human lactoferrin. Plaques formed by the infecting virus were visualized by crystal violet staining after 5 days of culture in the presence of human immunoglobulin (a-c) or by immunoperoxidase staining with polyclonal anti-HSV-1 antibody after 20 h of culture (d). Lactoferrin dramatically impaired the cell-to-cell spread of HSV-1 KOS and gC^-^39, but not of Rid1.

HLf was furthermore found to efficiently inhibit HSV-1 cell-to-cell spread as observed by crystal violet staining five days post-infection and by IPS 20 h.p.i. (Figure 5.). This observation corroborates with previous observations [5-7]. In the current study, the inhibition was highly virus strain-dependent. Cell-to-cell spread of wt and gC^-^39 viruses, but not of Rid1, was dramatically impaired at hLf concentration of 500 μg/ml independently of the degree of hLf iron-saturation (Figure 5). Some inhibition was observed already at a concentration of 250 μg/ml, but not in lower concentrations (data not shown). Cell-to-cell spread is known to involve primarily gB and gE/gI and also gD in certain cell types [20,21], but not gC directly [22-24]. The susceptibility of gC-negative HSV-1 strain, gC^-^39, to inhibition of cell-to-cell spread may result from hLf interference with gB binding to heparan sulphate at cell junctions [24]. More importantly, the fact that the plaque-size of Rid1, with a modified gD, was not affected by hLf, suggests that the observed inhibition is dependent on gD or its cellular counterparts. Earlier, inhibition of HSV-1 cell-to-cell spread by lactoferricin, a peptide generated from Lf by pepsin cleavage, has been shown to involve chondroitin sulphate [7]. It is possible, that in addition to inhibiting cell-to-cell spread of HSV-1, hLf localized to the cell surface may also modulate the infectivity of the released progeny virus.

In conclusion, we found hLf to interfere with the progression of HSV-1 infection at multiple steps of the viral replication cycle and with HSV-1 cell-to-cell spread in a dose- and virus strain-dependent manner. Our studies show for the first time that in addition to inhibiting the adsorption and post-attachments steps of HSV-1 infection, hLf is also able to neutralize HSV-1 and that the inhibition of cell-to-cell spread is at least partly dependent on gD or its cellular counterparts. The degree of hLf iron-saturation played little role in the inhibition. HLz displayed no anti-HSV-1 activity, suggesting that cationic property alone has no significant role in the anti-HSV activity of a polypeptide of the mucosal innate defence system.

## Competing interests

The authors declare that they have no competing interests.

## Authors' contributions

HV participated in the design of the study, carried out the experiments and drafted the manuscript. VH, MW and JT have participated in the design of the study and helped drafting the manuscript. All authors read and approved the final manuscript.
